# The Relationship of CSF and Plasma Cytokine Levels in HIV Infected Patients with Neurocognitive Impairment

**DOI:** 10.1155/2015/506872

**Published:** 2015-03-03

**Authors:** Lin Yuan, An Liu, Luxin Qiao, Bo Sheng, Meng Xu, Wei Li, Dexi Chen

**Affiliations:** ^1^Center for Infectious Disease, Beijing Youan Hospital, Capital Medical University, Beijing 100069, China; ^2^Beijing Institute of Liver Disease, Beijing 100069, China

## Abstract

Although HAD is now rare due to HAART, the milder forms of HAND persist in HIV-infected patients. HIV-induced systemic and localized inflammation is considered to be one of the mechanisms of HAND. The levels of cytokines in CSF were associated with neurocognitive impairment in HIV infection. However, the changes of cytokines involved in cognition impairment in plasma have not been shown, and their relationships between CSF and plasma require to be addressed. We compared cytokine levels in paired CSF and plasma samples from HIV-infected individuals with or without neurocognitive impairment. Cytokine concentrations were measured by Luminex xMAP. In comparing the expression levels of cytokines in plasma and CSF, IFN-*α*2, IL-8, IP-10, and MCP-1 were significantly higher in CSF. Eotaxin was significantly higher in plasma, whereas G-CSF showed no difference between plasma and CSF. G-CSF (*P* = 0.0079), IL-8 (*P* = 0.0223), IP-10 (*P* = 0.0109), and MCP-1 (*P* = 0.0497) in CSF showed significant difference between HIV-CI and HIV-NC group, which may indicate their relationship to HIV associated neurocognitive impairment. In addition, G-CSF (*P* = 0.0191) and IP-10 (*P* = 0.0377) in plasma were significantly higher in HIV-CI than HIV-NC. The consistent changes of G-CSF and IP-10 in paired plasma and CSF samples might enhance their potential for predicting HAND.

## 1. Introduction

HIV associated neurocognitive disorder (HAND) is a prevalent and significant challenge to HIV infected populations [1]. Its clinical severity includes asymptomatic neurocognitive impairment (ANI), mild neurocognitive disorder (MND), and more severe HIV associated dementia (HAD) [2]. The introduction of highly antiretroviral therapy (HAART) has effectively reduced the mortality and morbidity related to HIV; however, the overall prevalence of HAND remains high. More than 50% of HIV infected persons receiving HAART demonstrate milder HAND syndromes [1]. Progressive neurocognitive disorders in HIV patients are related with impaired quality of life [3–6], poorer antiretroviral adherence [7–11], and higher mortality [12]. The pathogenic mechanism of HAND has not been clear. Inflammatory response in the periphery and central nervous system (CNS) where monocyte/macrophage (M/M) is activated has been correlated with HAND [13, 14]. Inflammatory factors and chemokines expressed by activated M/M play a critical role in neuroinflammation and neurodegeneration in HAND [15–17]. In our previous study we demonstrated the elevation of a small panel of cytokines in CSF was correlated with neurocognitive impairment in HIV infected patients [18]. However, there is still lack of data addressing the relationship between blood and CSF concentrations of cytokines in relation to HAND.

Thus, this study aimed to firstly determine whether CSF levels of the inflammatory cytokine or chemokine were elevated in HIV infected patients with neurocognitive impairment and secondly to examine relationships between the concentrations of these cytokines in the CSF and plasma.

## 2. Methods

### 2.1. Study Participants

HIV infected patients were enrolled in Henan and Yunnan provinces in China, and CSF were acquired at the time of a clinically indicated lumbar puncture. CSF and plasma samples were taken simultaneously. All the samples were centrifuged, and cell-free CSF and plasma were aliquot and immediately frozen at −80°C. The Memorial Sloan-Kettering Scale (MSK) was used to categorize the neurocognitive impairment of each patient. This study was approved by ethics committees at the Capital University of Medical Science in Beijing, and all participants provided informed consent.

### 2.2. Measurement of CSF Cytokines

A total of 6 cytokines were measured in paired CSF and plasma samples using human cytokine/chemokine MILLIplex kits (Millipore Corp, Billerica, MA, USA): IL-8, eotaxin, granulocyte colony-stimulating factor (G-CSF), interferon- (IFN-) *α*2, IFN-*γ*-induced protein- (IP-) 10, and monocyte chemotactic protein- (MCP-) 1. The lower levels of detection were ranged between 0.2 and 10.1 pg/mL for each of the cytokines measured. All samples were assayed concurrently in duplicate. Data were collected using a Bio-Plex Suspension Array Reader (Bio-Rad Laboratories Inc., Hercules, California, USA). Cytokine concentrations that were lower than the lower limits of detection were reported as undetectable (0).

### 2.3. Statistical Analyses

Univariate analyses were performed using GraphPad Prism 5 (GraphPad Software, San Diego, California, USA). Mann-Whitney *U* test and Wilcoxon signed rank test were used for unmatched and matched comparisons, respectively. Spearman rank tests were used to test for correlations. *P* values less than 0.05 were considered significant.

## 3. Results

There were 85 HIV-1 clade B or B′ infected patients enrolled with paired CSF and plasma samples collected for cytokine analysis. The median age was 38 years (range, 11–76 years) and 59 (69%) were men. The risk factors for HIV infection were sexual transmission (*n* = 44), intravenous drug use (*n* = 15), blood transfusion (*n* = 7), paid blood or plasma donation (*n* = 2), mother-to-child transmission (*n* = 2), and unknown (*n* = 15). In total, 29 of 85 patients had complications, including cryptococcal meningitis (*n* = 21), tuberculous meningitis (*n* = 2),* Toxoplasma* encephalitis (*n* = 2),* Pneumocystis *pneumonia (*n* = 2), and cytomegalovirus radiculitis (*n* = 2). Nearly half of the HIV infected patients (43%) were receiving HAART at the time of evaluation. All were on multidrug combination ART regimens, which consisted of at least 2 NRTIs (e.g., AZT, D4T, 3TC, DDI, and TDF) plus an NNRTI (NVP or EFV) or a PI (LPV). None were on mono- or dual-therapy. Based on MSK classification, patients were classified into HIV infected with normal cognition (HIV-NC; *n* = 33, MSK = 0) and impaired cognition groups (HIV-CI; *n* = 52). The HIV-CI group consisted of MSK = 0.5 (*n* = 24), MSK = 1 (*n* = 12), MSK = 2 (*n* = 10), and MSK = 3 (*n* = 6). The viral burden in CSF or plasma of HIV-CI group was higher than that of HIV-NC group, accompanied with lower CD4 cell count. However, there was no statistically significant difference whether in viral load or in CD4 cell count for two groups (Table 1).

### 3.1. Plasma and CSF Cytokines

**Figure 1:**
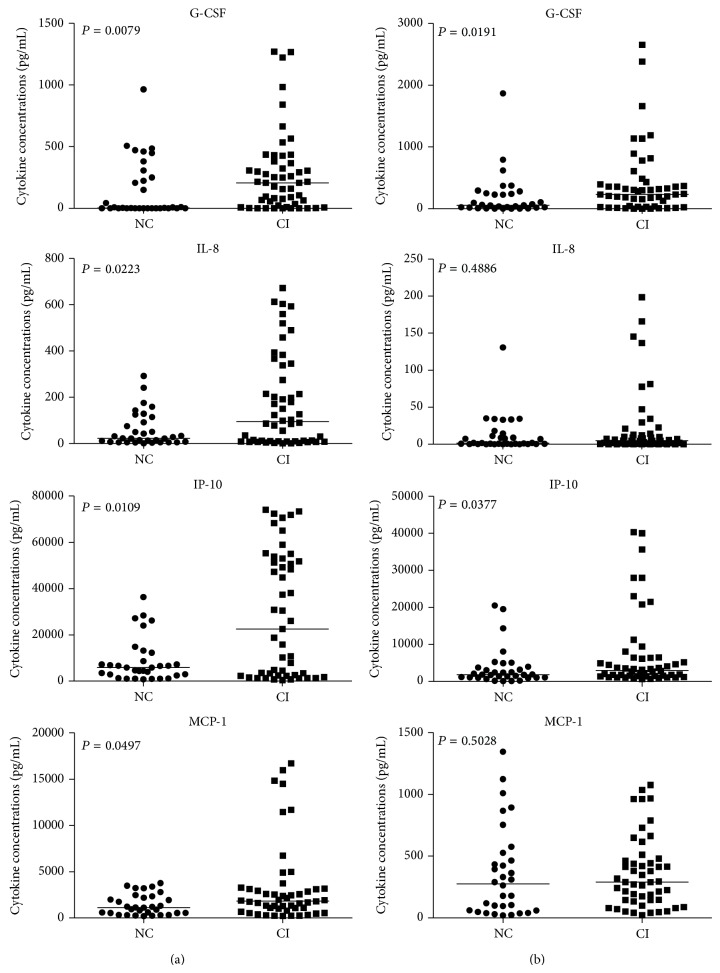
Comparison of G-CSF, IL-8, IP-10, and MCP-1 concentrations in paired CSF and plasma samples from 33 HIV infected subjects with normal cognition (HIV-NC) and 52 HIV infected subjects with cognition impairment (HIV-CI). Values below the lower limit of detection of the assay are reported as zero. The levels of G-CSF, IL-8, IP-10, and MCP-1 in CSF from HIV-CI group were significantly higher than HIV-NC group (a), whereas in plasma samples, only G-CSF and IP-10 showed significant difference (b). Differences were analyzed by the Mann-Whitney *U* test. *P* values of <0.05 were considered significant. Dots, cytokine level in CSF for each study subject; horizontal lines, median values for each group.

HIV infected patients with neurocognitive impairment had elevated levels of cytokines in CSF and plasma in comparison to those with normal neurocognition (Table 1). There was significant difference in CSF levels of G-CSF (*P* = 0.0079), IL-8 (*P* = 0.0223), IP-10 (*P* = 0.0109), and MCP-1 (*P* = 0.0497) between HIV-CI and HIV-NC group (Figure 1(a)). However, in plasma only G-CSF (*P* = 0.0191) and IP-10 (*P* = 0.0377) showed significant difference (Figure 1(b)). The concentrations of eotaxin and IFN-*α*2 did not show any significant difference between these two groups either in CSF or in plasma (data not shown).

### 3.2. Relationships between Cytokine Concentrations in CSF and Plasma

In HIV infected patients, the expression levels of IFN-*α*2, IL-8, IP-10, and MCP-1 in CSF were significantly higher than in plasma. In contrast, eotaxin was significantly higher in plasma. But there was no difference for G-CSF. The expression levels of several cytokines showed significant correlations between CSF and plasma. Among them IP-10 presented the strongest correlation in the patients with HIV-CI (correlation coefficient 0.7525, *P* < 0.0001) (Table 2).

## 4. Discussion

We demonstrated that the concentrations of G-CSF, IL-8, IP-10, and MCP-1 in CSF samples from neurocognitive impaired HIV-1 infected patient were significantly higher than patients with normal cognition. This confirmed our finding in previously published study [18]. Elevated concentrations of cytokines that we examined reflected the activation of the immune response in central nervous system or periphery, which might provide a cytokine panel for predicting neurocognitive impairment in HIV-1 infected individuals. Clinical studies on the role of inflammation in HIV-1 associated neurocognitive disorder rely on determinations of biomarkers in clinically accessible fluid compartments. CSF sample is not more likely to reach as plasma sample. In this study, there was not any correlation between paired CSF and plasma concentrations for any of the cytokines involved. This might indicate a limitation of plasma samples in reflecting the immune response in CNS. Fortunately, we found the concentrations of G-CSF and IP-10 in plasma of HIV-CI patient were higher than HIV-NC patients. The advantages of plasma sample, such as being most accessible and being able to be measured repeatedly, will enhance the potential of plasma G-CSF and IP-10 for predicting HIV-1 related cognitive impairment. Additionally, the site of the highest cytokine level for each cytokine was various in plasma or CSF. IL-8, IFN-*α*2, IP-10, and MCP-1 were higher in CSF than plasma samples, whereas eotaxin and G-CSF were significantly higher in plasma. Taking into account the lack of correlation between CSF and plasma cytokine concentrations, these might suggest that some cytokines may be localized within the CSF which is the potential origin of immune response rather than resulting from the dissemination of cytokines via blood stream.

Interferon-*γ*-inducible protein 10, IP-10 (in the systematic nomenclature, CXCL10), is expressed by astrocytes, microglia, and endothelial cells in an inflammatory lesion and cells culture supernatants, acts specifically on activated T cells and macrophages, and attracts T lymphocytes into the CSF. Expression of IP-10 in the brain is correlated closely with increased infiltrating cells in herpes simplex virus (HSV), MHV, and West Nile virus (WNV) infection [19–21]. Abrogation of IP-10 expression by either depletion with anti-IP-10 or genetic silencing dramatically reduces infiltration of T cell into the CNS. In the brain, IP-10 can be induced by HIV-1 viral gp120, Nef, and Tat [22–24]. Cooperative interaction of HIV Tat and IFN-*γ* results in IP-10 overexpression, which in turn can amplify the inflammatory responses within the CNS of HAD patients by recruiting more lymphocytes into the brain. IP-10 has been detected in the CSF of individuals with HIV-1 infection [25] and in the astrocytes in brains of individuals with HIV associated dementia [26]. It was also associated with SHIV encephalitis in macaques [27]. Upregulation of IP-10 may contribute to the BBB permeability changes and instruction of innate immune responses in CNS [28]. Zhang et al. indicated that IP-10 could intensify injury to the blood-brain barrier by NK cells via its receptor CXCR3 [29]. Exogenous CXCL10 led to increased membrane permeability in neurons and caused cell loss in the fetal brain cultures, which might be responsible for induction of neuron apoptosis [30]. In our study, we demonstrated the expression of IP-10 was significantly elevated in HIV-1 infected patients with neurocognitive impairment whether in CSF or in plasma. In addition, the expression levels of IP-10 in CSF and plasma in the patients with HIV-CI were significantly correlated (correlation coefficient 0.7525, *P* < 0.0001), but there was no correlation in HIV-NC patients. It suggested that neurocognitive impairment in HIV-1 infected patients might be accompanied by the arising of blood-brain barrier (BBB) impairment. Increased permeability of BBB results in infiltration of activated T lymphocyte from periphery and overexpression of cytokines in CNS, which might promote the degeneration of neuron. Further studies on relationship between IP-10 and BBB injury are required.

There are some limitations to our study. Firstly, cases with HIV complication have not been excluded from the enrolled subjects (29/85), which might raise the possibility that an increase in cytokine levels with complications could explain their higher values. However, the ratios of cases with complication were almost equal in HIV-CI and HIV-NC groups, with 33% and 36%, respectively, making this explanation unlikely. Secondly, the patients in this study have relative lower CD4 T cell count [53 (20–120), median (IQR)]. There was no significant difference in CD4 cell count between HIV-CI and HIV-NC patients; nevertheless, relative poorer immune state could exert a negative influence on inflammatory response.

In conclusion, our study suggested that elevated levels of G-CSF, IL-8, IP-10, and MCP-1 in CSF may indicate HIV associated neurocognitive impairment. Consistent changes of G-CSF and IP-10 in paired plasma and CSF samples enhance their potential for predicting HAND.

## Figures and Tables

**Table 1:** Demographic and disease characteristics of subjects.

Characteristics	HIV-CI	HIV-NC	*P* value
Number of subjects	52	33	
Age in years [median (IQR)]	38 (31–42)	38 (33–42)	
Gender [*n* (%)]			
Male	38 (73%)	21 (64%)	
Female	14 (27%)	12 (36%)	
HIV RNA (log⁡10 copies/mL) [median (IQR)]			
CSF	4.50 (2.95–7.50)	3.06 (1.70–3.60)	*P* = 0.3640
Plasma	5.915 (3.44–7.50)	3.905 (2.70–5.21)	*P* = 0.8194
CD4+ cell count (cell/*μ*L) [median (IQR)]	41 (21–120)	69 (21–120)	*P* = 0.3275
CSF			
Cell count (cell/*μ*L) [median (IQR)]	12 (6–21)	11 (7–30)	*P* = 0.7499
Protein (mg/mL) [median (IQR)]	74 (73–110)	41 (34–48)	*P* = 0.0097
Number of threated by HAART [*n* (%)]	20 (38.5)	17 (51.5)	
Treatment duration (months) [median (range)]	4 (2–6)	6 (2–18)	
CNS complications [*n* (%)]	17 (33%)	12 (36%)	

**Table 2:** Cytokine concentrations in paired CSF and plasma samples.

	HIV-CI				HIV-NC			
	Plasma	CSF	Wilcoxon signed rank, *P*	Correlation coefficient^*^	Plasma	CSF	Wilcoxon signed rank, *P*	Correlation coefficient^*^
Eotaxin								
Median	78.86	0.94	<0.0001	−0.3949	59.36	0.79	0.0019	−0.6241
IQR	15.88–202.3	0–45.9		*P* = 0.0038	6.54–200.4	0–1.165		*P* = 0.0001
<Detection Limit	2%	56%			0%	37%		
G-CSF								
Median	234.8	207	0.4255	0.1667	64.3	4.36	0.2436	0.5353
IQR	31.54–421.5	26.53–415.2		*P* = 0.2375	17.62–287.5	0–279.2		*P* = 0.0013
<Detection Limit	4%	13%			8%	27%		
IFN-*α*2								
Median	29.03	66.79	0.0039	0.6903	10.82	40.58	0.0174	0.4831
IQR	5.59–80.2	0.74–175.7		*P* < 0.0001	0–55.05	0–104.6		*P* = 0.0044
<Detection Limit	10%	25%			23%	17%		
IL-8								
Median	4.78	100.6	<0.0001	−0.07862	1.47	22.44	<0.0001	0.1896
IQR	0.71–13.82	8.86–343.4		*P* = 0.5796	0.375–12.82	5.92–102.4		*P* = 0.2907
<Detection Limit	29%	4%			29%	2%		
IP-10								
Median	2985	17260	<0.0001	0.7525	1845	5834	0.001	0.1756
IQR	1451–6428	1828–51620		*P* < 0.0001	1046–3891	2521–11330		*P* = 0.3364
<Detection Limit	0%	6%			0%	2%		
MCP-1								
Median	292.1	1755	<0.0001	0.1527	289.4	1120	<0.0001	0.08761
IQR	144–502.9	692.9–3147		*P* = 0.2798	60.25–550.8	526.9–2294		*P* = 0.6335
<Detection Limit	0%	4%			0%	2%		

^*^Spearman rank correlation, values below the lower limits of detection set at zero.
